# An Introductory Lecture to the Course of Institutes of Medicine, &c., Delivered in Jefferson Medical College, November 1, 1841

**Published:** 1841-12-11

**Authors:** 


### MEDICAL EXAMINER.

DEVOTED TO MEDICINE, SURGERY, AND THE COLLATERAL SCIENCES.

No. 50.] PHILADELPHIA, SATURDAY, DECEMBER 11, 1841. [Vol. IV.

### BIBLIOGRAPHICAL NOTICE.

*An Introductory Lecture to the Course of Institutes of Medicine, &c., delivered in Jefferson Medical College, November* 1, 1841. By Professor Dunglison.

The Introductories are multiplying so thickly upon our hands, that we are compelled to curtail the limits that we have heretofore allowed ourselves in noticing them. The veteran professor and literateur whose lecture is before us may be fairly disposed of in brief terms—an old familiar, whose merits are appreciated, and need not be enlarged upon. Professor Dunglison's lecture was, we believe, the first of the course at the Jefferson College; and, as the connecting link between the old and new organization of the school, upon him devolved the duty of welcoming his new associates, and adverting to the circumstances under which the former faculty was broken up. The Professor naturally and appropriately felicitates the institution upon the accession of strength which it has received under the new organization. Collectively, he next compliments his colleagues upon their eminent qualifications, and expatiates upon the "extensive facilities," "preparations, specimens, plates, drawings, and apparatus" which are in the hands of the institution. Professor Dunglison has too much good taste to indulge in immediate self-laudation; his brief allusion to the claims of his school on the consideration of the profession he justifies, on the plea of its peculiar position, and the example of sister institutions; particularly the elder sister, who "has been engaged in the business of medical instruction for three-quarters of a century." The plea is a fair one. The custom, however, we cannot help thinking more honoured in the breach than the observance. Let us not be understood to apply the force of this remark to the lecturer of whom we have been speaking. If at all, he has sinned most gently, and to his course of undeviating propriety, in and out of the chair, all bear testimony. But we do address our decided, if feeble, censure to the system of puffing, direct and indirect, so freely resorted to by some of the medical schools. It is unworthy the dignity of the profession, and if it accomplish temporary purposes, can never win lasting and elevated reputation.

The subject proper of Dr. Dunglison's lecture, a somewhat trite theme—the progress of medical science—he has handled with the taste and ability that belongs to the effusions of his practised pen. New and apposite in his illustrations, neat and flowing in style, he has worked up an exceedingly readable sketch of this pretty threadbare subject. While of course a topic like this, common place as it may prove to the general reader, is very appropriately selected for discussion as an introductory lecture, Dr. Dunglison has done more than common justice to the subject.

